# Temperature-Dependent Models for Rutting Performance of Asphalt Pavement Surface Layer Materials Under Varying Load Conditions

**DOI:** 10.3390/ma18204708

**Published:** 2025-10-14

**Authors:** Jincai Yang, Guanqing Li, Yantao Chen, Zhentao Yan, Yue Wang, Shenghan Zhuang, Yingjun Jiang

**Affiliations:** 1 Nanyang Communications Construction Investment Group Co., Ltd., Nanyang 473000, China; jincaiyang2025@126.com; 2 Nanyang Fangzao Highway Co., Ltd., Nanyang 473000, China; guanqingli0908@126.com (G.L.); yantaochen126@126.com (Y.C.); 3 School of Highway, Chang’an University, Xi’an 710064, China; 2025221315@chd.edu.cn (Z.Y.); 2023021084@chd.edu.cn (Y.W.); 4 Department of Bridge Engineering, Southwest Jiaotong University, Chengdu 610031, China; shenghanzhuang@my.swjtu.edu.cn

**Keywords:** asphalt mixture, surface layer, temperature, rutting deformation, dynamic stability

## Abstract

In order to accurately characterize the temperature dependence of the high-temperature performance of asphalt pavement surface layer materials, this paper studies the effects of temperature, load, and number of actions on the high-temperature anti-rutting performance of asphalt pavement surface layer materials (AC-13 and AC-16 mixtures), based on indoor rutting tests, and constructs a high-temperature performance temperature-dependent model and rutting prediction model for surface layer materials. The results show that as the temperature increases, the dynamic stability of the mixture decreases in an S-shaped curve, and the rut depth increases exponentially. The temperature-dependent model transition temperatures for the dynamic stability of 70#, 50#, 30#, SBS, and HMB asphalt mixtures are 39 °C, 44 °C, 46 °C, 56 °C, and 56 °C, respectively. The dynamic stability of modified asphalt mixtures is significantly higher than that of base asphalt mixtures. The depth of wheel ruts is affected by temperature, load, and the number of actions. The variation ranges of the load index *k_P_*, the temperature index *k_T_*, and the number of actions index *k_N_* are 0.727–1.222, 1.926–2.177, and 0.133–0.295, respectively. The correlation coefficients of the wheel rut prediction model are all above 0.95, and the depth of wheel ruts can be predicted by the model.

## 1. Introduction

In the context of global warming, data concerning extremely high temperatures worldwide are being updated with increasing frequency, with occurrences of temperatures exceeding 40 °C becoming notably prevalent and occasionally approaching approximately 45 °C [1,2]. These temperature extremes have profound implications for the utilization and performance of road traffic infrastructure [3]. The surface layer of asphalt pavement, owing to its direct exposure to environmental conditions, is particularly susceptible to the effects of elevated temperatures [4,5]. Empirical evidence demonstrates that the surface temperature of asphalt pavement can increase by 15 to 25 degrees Celsius above ambient temperatures. Consequently, when ambient temperatures surpass 40 °C, the pavement surface temperature may reach between 65 and 75 °C, thereby significantly elevating the risk of pavement distress, such as rutting, deformation, and diminished load-bearing capacity [6]. Furthermore, research identifies temperature as the principal factor contributing to non-load-related distress in asphalt pavements [7,8,9]. When the internal temperature of asphalt pavement exceeds the asphalt’s softening point, the mixture’s fluidity increases, resulting in heightened shear stress within the pavement structure and consequently increasing the likelihood of rutting damage [10,11]. The combination of elevated temperature and excessive loading markedly intensifies the risk of damage to the asphalt mixture, culminating in pronounced rutting deformation [12,13]. Comparative studies indicate that, relative to conventional base asphalt, SBS-modified asphalt, and rubber asphalt, high-viscosity asphalt and lower-grade asphalt demonstrate superior resistance to high-temperature rutting [14,15]. Moreover, the permanent deformation of asphalt mixtures escalates with increasing load and temperature, with the synergistic effects of these factors adversely affecting the mixture’s deformation resistance [16,17].

To quantitatively evaluate the influence of various factors on the high-temperature rutting resistance of asphalt mixtures and to predict rutting progression, Mu et al. [18] conducted prolonged rutting tests on three distinct asphalt mixture types across a spectrum of temperature conditions. This methodology facilitated the derivation of temperature-dependent rutting rates that more precisely represent the high-temperature stability of the mixtures. Shang et al. [19] examined variations in dynamic stability and rutting deformation of interlayer mixtures subjected to different temperature regimes and asphalt binder types. Additionally, they developed a temperature-dependent model to characterize rutting behavior, thereby providing a scientific basis for optimizing asphalt pavement design and enhancing rutting resistance. Javilla et al. [20] carried out rutting experiments on asphalt mixtures to investigate the effects of multiple stress loads on rutting development, subsequently formulating a multi-stress analysis model and establishing pertinent rutting performance indicators. Through rutting and dynamic modulus testing, Sun et al. [21] demonstrated that high-modulus asphalt mixtures possess superior resistance to deformation, rutting, and high-temperature instability, while exhibiting low sensitivity to variations in traffic loading. Xu et al. [22] explored the anti-rutting performance and underlying mechanisms of three different asphalt mixtures under varying high-temperature and load conditions, finding a linear decrease in anti-rutting performance across all mixtures with increasing temperature and load. Li et al. [23] analyzed the impact of rutting progression on the high-temperature performance of individual asphalt pavement layers. Guo et al. [24] investigated factors influencing the rutting resistance of asphalt pavements, concluding that elevated temperatures and load levels correspond to diminished rutting resistance in asphalt mixtures. Zhang et al. [25] assessed the effects of climate change on rut recovery capacity in selected representative cities, revealing that failure to incorporate climate change considerations in asphalt pavement rutting design leads to insufficient recovery capabilities. Zhao et al. [26] proposed a robust methodology for accurately predicting the rutting resistance of asphalt mixtures and pavement surfaces by integrating laboratory experiments with finite element modeling techniques. Huang et al. [27] developed a viscoelastic–plastic damage constitutive model tailored for asphalt mixtures to evaluate permanent deformation under cyclic loading. Their results indicated that cumulative deformation of the asphalt layer increased progressively with the number of loading cycles, although the rate of increase diminished over time.

The studies reviewed have substantially enhanced the comprehension of factors influencing the high-temperature behavior of asphalt mixtures. Nonetheless, existing predictive models in the literature typically address only one or two key variables—namely, temperature, load magnitude, or loading cycles—while a comprehensive constitutive model that concurrently integrates all three factors remains lacking. Additionally, the characteristic sigmoidal decline in dynamic stability as a function of temperature has not been sufficiently captured by traditional functional forms. To address these gaps, the present study makes two significant contributions that set it apart from prior research. Firstly, it introduces an innovative application of the Boltzmann function to precisely characterize the temperature dependence of dynamic stability, thereby facilitating accurate identification of performance transition temperatures across various asphalt types. Secondly, it develops a unified rutting prediction model that holistically incorporates the interactive effects of temperature, load magnitude, and the loading number. This integrated framework offers a more robust predictive tool and yields enhanced insights into the performance of asphalt mixtures under complex operational conditions.

## 2. Research Approach

### 2.1. Raw Material

#### 2.1.1. Asphalt

The asphalt materials used in this study included 70# base asphalt from Jingbo Corporation, Binzhou, China. 50# and 30# base asphalt from Jiangsu Tiannuo Road Materials Co., Ltd., Zhenjiang, China. SBS-modified asphalt from Maoming Xinlu Building Materials Technology Co., Ltd. Maoming, China, and HMB high-modulus asphalt supplied by Zhongli Company, Pinghu, China. Their technical specifications are listed in Table 1 and Table 2, respectively.

#### 2.1.2. Mineral Aggregate

(1) Coarse Aggregate

The coarse aggregates used are basalt crushed stones produced by Shaanxi Rongxin Mining Development Co., Ltd., Shangluo, China. Their specifications are 16–19 mm, 13.2–16 mm, 9.5–13.2 mm, and 4.75–9.5 mm. The technical properties of each specification of aggregates are shown in Table 3.

(2) Fine Aggregate

The fine aggregate is made from limestone manufactured sand produced by Shanxi Rongxin Mining Development Co., Ltd., Shangluo, China. Its technical properties are shown in Table 4.

(3) Mineral Powder

The mineral powder is made from limestone produced by Shanxi Rongxin Mining Development Co., Ltd., Shangluo, China. Its technical properties are shown in Table 5.

### 2.2. Research Approach

The types of asphalt adopted were 70# base asphalt, 50# base asphalt, 30# base asphalt, SBS-modified asphalt, and HMB high-modulus asphalt.

The mineral aggregate gradation included an AC-13 mixture and an AC-16 mixture. The test items included track depth (*RD*) and dynamic stability (*DS*). The test load was as follows: 0.5 MPa, 0.7 MPa, 0.9 MPa, and 1.1 MPa. The test temperature was as follows: 15 °C, 30 °C, 45 °C, 60 °C, 65 °C, and 75 °C.

### 2.3. Experimental Procedure and Specifications

The rutting tests were performed in strict compliance with the Chinese standard “Standard Test Methods of Bitumen and Bituminous Mixtures for Highway Engineering (JTG E20-2011)” [28]. The detailed methodology is outlined as follows:

Temperature Control and Monitoring: Specimens were conditioned within a temperature-controlled chamber until a uniform and stable temperature was achieved throughout the entire volume, a condition verified prior to the initiation of loading. The chamber maintained the target test temperature with a precision of ±0.5 °C. The actual temperature of each test specimen was continuously monitored and confirmed using calibrated thermometers or thermocouples.

Load Application Simulation: Repeated loading was simulated employing a standard automatic wheel tracking tester, Beijing Aerospace Keyu Test Instrument Co., Ltd., Beijing, China. The standard contact pressure was set at 0.7 MPa with a tolerance of ±0.05 MPa. For the purposes of this investigation, additional load levels of 0.5 MPa, 0.9 MPa, and 1.1 MPa were also applied. The loading wheel reciprocated along a fixed path on the specimen surface at a frequency of 42 passes per minute. The total number of load cycles was automatically recorded by the data acquisition system integrated within the testing apparatus.

Specimen Conditioning: Specimens were placed in the environmental chamber, Beijing Aerospace Keyu Test Instrument Co., Ltd., Beijing, China. at the designated test temperature for a minimum duration of five hours to ensure thermal equilibrium throughout each specimen. This procedure was implemented to eliminate internal thermal gradients that could potentially compromise the accuracy and reliability of the test outcomes.

## 3. Influence of Test Temperature on the Dynamic Stability of Asphalt Pavement Surface Layer Materials

**Figure 1:**
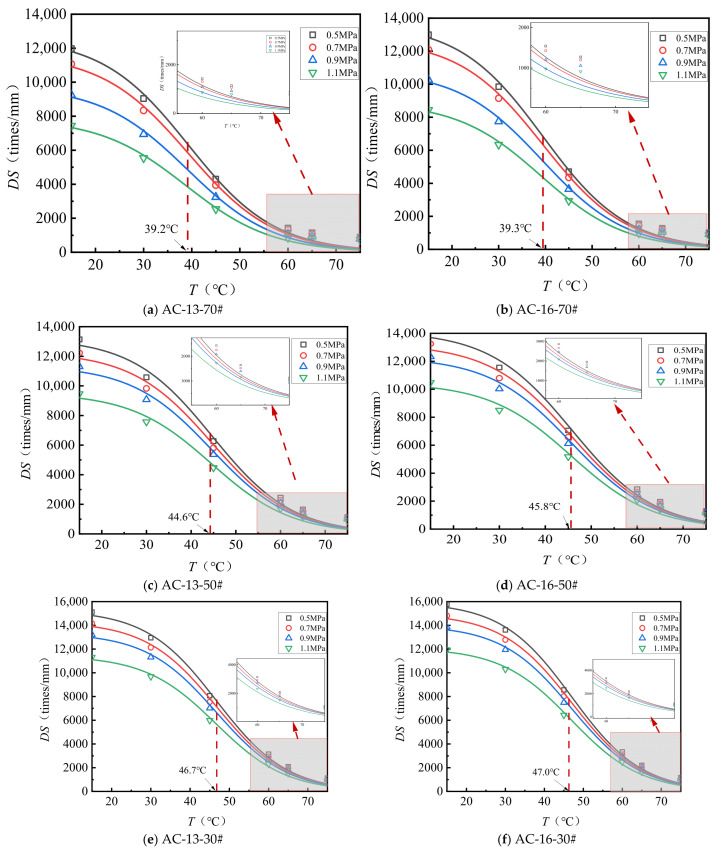

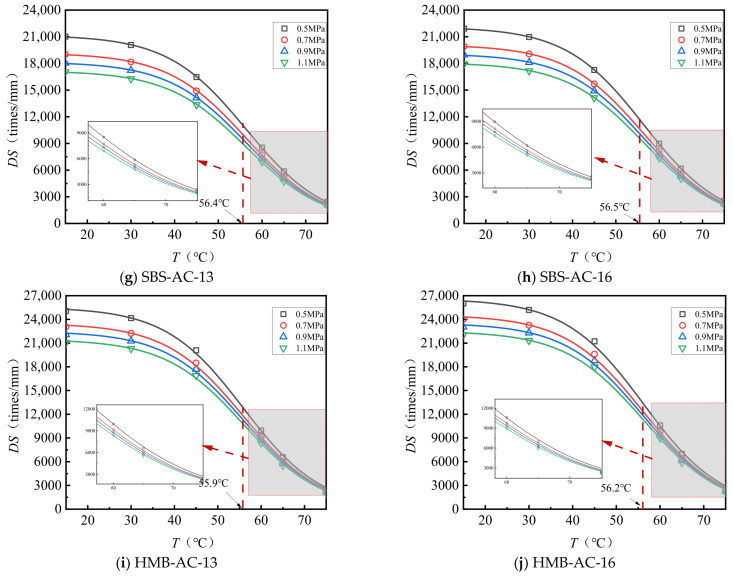
Relationship between *DS* and *T* of surface layer materials.

Figure 1 illustrates the dynamic stability of the asphalt surface layer material under various test conditions, while Table 6 presents the relative values of maximum dynamic stability, using 1.1 MPa dynamic stability as the reference. The specimen dimensions are 300 mm × 300 mm × 50 mm.

As shown in Figure 1 and Table 6, (1) temperature significantly influences the mixture’s dynamic stability. As temperature increases, dynamic stability decreases following an S-shaped curve. At 15 °C, dynamic stability can be more than ten times higher than at 75 °C. (2) Lower temperatures amplify the effects of load and nominal maximum particle size on dynamic stability. At 15 °C, dynamic stability decreases markedly with increasing load, whereas at 75 °C, mixtures with the same asphalt type and mineral gradation tend to stabilize at a certain dynamic stability value. (3) At 15 °C, under a 0.5 MPa load, the dynamic stability of the AC-13 and AC-16 mixtures ranges from 1.19 to 1.61 times and 1.18 to 1.54 times, respectively, compared to that under 1.1 MPa. The 70# asphalt mixture shows greater sensitivity to load at 15 °C, while the HMB mixture is the least sensitive under the same conditions.

To guarantee the reliability and reproducibility of the experimental outcomes, each rutting test was performed with a minimum of three replicates under each testing condition. The coefficient of variation (*C_v_*) was employed to evaluate the reliability of the test data, as shown in Table 7. *C_v_* is defined as the ratio of the standard deviation to the mean value, expressed as a percentage. A lower *C_v_* value signifies greater consistency and reduced variability among the replicate tests. In the present study, *C_v_* for all test data remained below 10%, thereby confirming the high reproducibility and reliability of the experimental results.

## 4. Research on the Temperature-Dependent Model of Dynamic Stability of Surface Layer Materials for Asphalt Pavement

From the relationship between dynamic stability and temperature, it can be shown that dynamic stability follows an inverse S-shaped curve trend, decreasing with temperature. Therefore, a temperature-dependent model of dynamic stability is proposed to be constructed using the Boltzmann function form. The fitting formula is shown in Equation (1), and the fitting parameters are presented in Table 8.

(1) $$DS=\frac{{DS}_{max}}{1+e^{\frac{T-T_{0}}{9}}}$$

where *DS* is the predicted dynamic stability, times/mm; *DS_max_* is the maximum dynamic stability, times/mm; *T*_0_ is the temperature at which the slope of the dynamic stability curve attains its maximum value, °C; and *T* is the testing temperature, °C.

As presented in Table 8, Equation (1) provides an effective representation of the relationship between dynamic stability and temperature. The observed decline in dynamic stability with increasing temperature occurs in three distinct phases. Under identical loading conditions, the maximum dynamic stability (*DS_max_*) values for various asphalt types, ranked from lowest to highest, are as follows: 70# < 50# < 30# < SBS < HMB. Notably, the *DS_max_* values for SBS and HMB are substantially greater than those of the base asphalt. For a given asphalt type, *DS_max_* decreases markedly with increasing load, with the reduction being more pronounced in the base asphalt compared to SBS and HMB. When considering the same asphalt type and mineral aggregate gradation, the transition temperatures of dynamic stability exhibit minimal variation across different load levels. The sequence of dynamic stability transition temperatures for the different asphalt types, from lowest to highest, is 70# < 50# < 30# < SBS < HMB. This transition temperature correlates with the asphalt’s softening point, such that a lower softening point corresponds to a lower transition temperature. SBS and HMB demonstrate the highest transition temperatures, indicating that mixtures incorporating these asphalts possess superior resistance to deformation under elevated temperature conditions.

**Figure 2:**
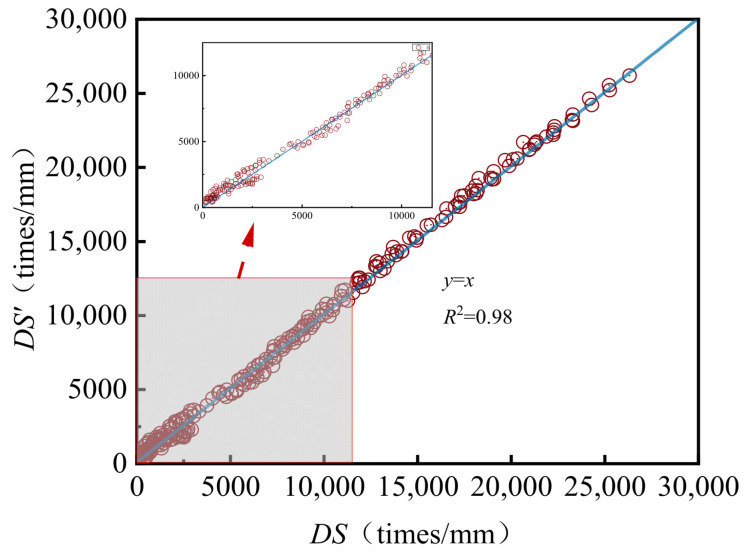
The relationship between *DS*’ and *DS*.

Figure 2 illustrates the relationship between the estimated dynamic stability values (*DS*’) and the experimentally measured values (*DS*). By substituting the fitted parameters into the corresponding fitting function, predicted dynamic stability values at various temperatures are calculated. These predicted values are then compared with the observed measurements to assess the accuracy of the model.

As shown in Figure 2, the measured values and the predicted values of the dynamic stability are both close to the *y* = *x* line, and the correlation coefficient reaches 0.98, which proves that the fitting accuracy of the temperature dependence of the dynamic stability using Equation (1) is quite high.

The parameter *T*_0_, obtained from the Boltzmann function, serves not merely as a fitting constant but as a critical indicator of the thermal susceptibility of asphalt binders. From a physical perspective, *T*_0_ denotes the pivotal transition temperature at which the asphalt binder experiences a fundamental rheological transformation, shifting from a glassy or viscoelastic solid state to a predominantly viscous fluid state. This transition is inherently associated with the binder’s compositional and structural characteristics. The observed ascending sequence of *T*_0_ values (70# < 50# < 30# < SBS < HMB) corresponds directly to increasing stiffness and enhanced elastic recovery properties of the binders. Asphalt binders with lower penetration grades (e.g., 30#) and polymer modifications (such as the SBS and HMB) exhibit more resilient internal structures that effectively resist thermal degradation, thereby preserving sufficient stiffness to maintain aggregate interlock at elevated temperatures. Accordingly, a higher *T*_0_ value indicates an expanded service temperature range for the asphalt mixture prior to experiencing a critical decline in dynamic stability, thus providing a quantitative criterion for material selection in high-temperature environments.

## 5. Factors Affecting Rutting Depth in Asphalt Pavement Surface Layer Materials

### 5.1. The Progression Behavior of Rut Depth in Surface Layer Materials of Asphalt Pavements

**Figure 3:**
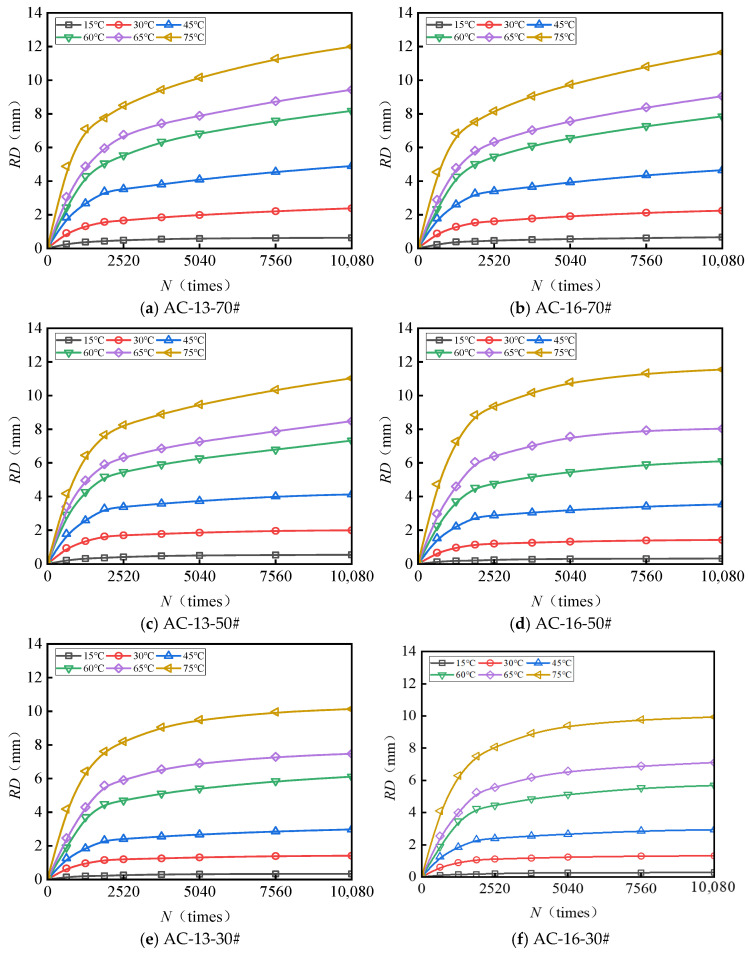

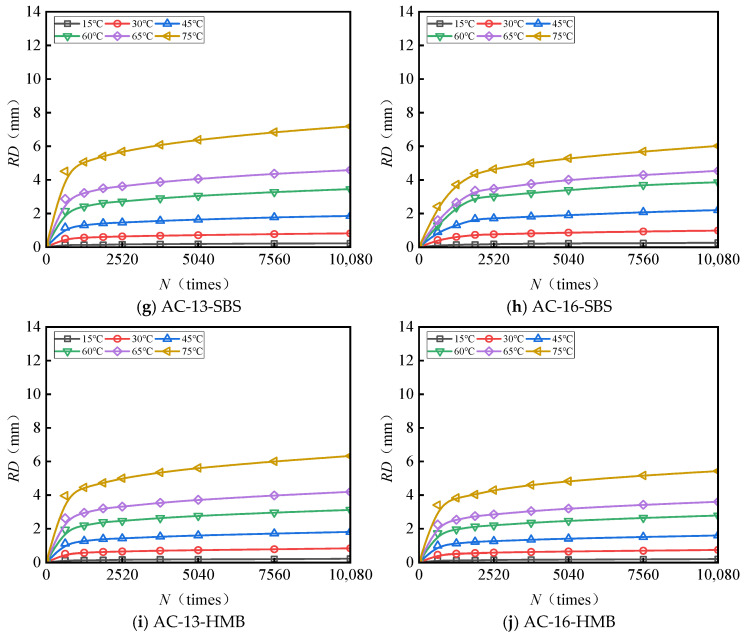
Variations in the rutting behavior of AC-13 and AC-16 subjected to a load of 0.7 MPa.

**Figure 4:**
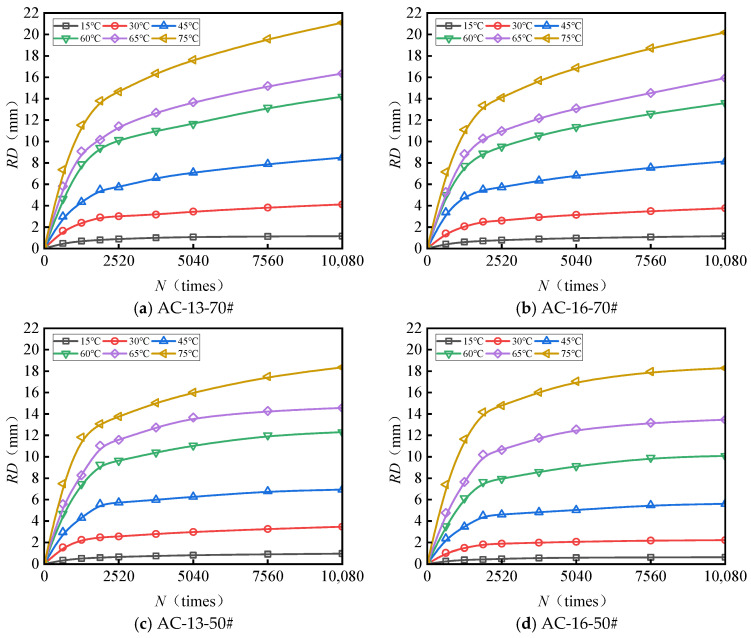

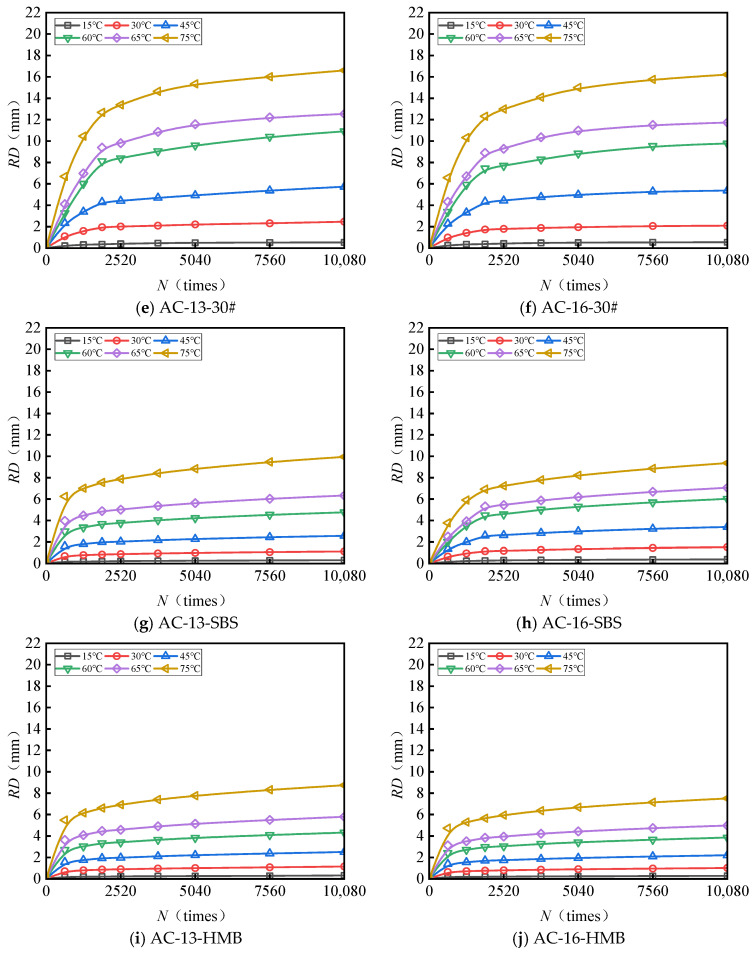
Variations in the rutting behavior of AC-13 and AC-16 subjected to a load of 1.1 MPa.

Figure 3 and Figure 4 present the test results pertaining to the rut depth progression characteristics of various surface layer materials.

Figure 3 and Figure 4 demonstrate that rut deformation escalates with increasing temperature (*T*), applied load (*P*), and the number of load cycles (*N*). Under consistent testing conditions, elevated temperatures significantly amplify rut depth. Likewise, higher applied loads correspond to a pronounced increase in rut depth, indicating that rut deformation is influenced by both temperature and load magnitude. The rut progression curve of the mixture reveals an initial phase characterized by rapid growth, followed by a subsequent stage of a more gradual and stable increase. After compaction to the standard density using a wheel roller, the mixture experiences additional compaction attributable to the rutting load. The rut deformation measured one hour prior to load application (equivalent to 42 × 60 = 2520 load cycles) is ascribed to compaction deformation. This suggests that before reaching 2520 load cycles, rut depth increases sharply, whereas beyond this threshold, rut deformation predominantly arises from shear deformation within the mixture.

### 5.2. Investigation into the Effect of Test Temperature on the Rutting Depth of Asphalt Pavement Surface Layer Materials

**Figure 5:**
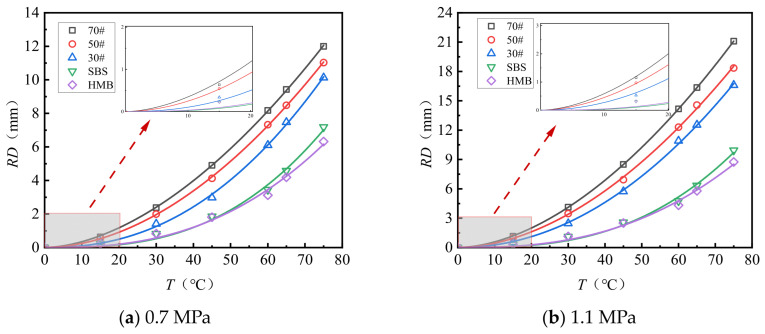
Temperature versus rutting deformation: curve fitting analysis (AC-13).

**Figure 6:**
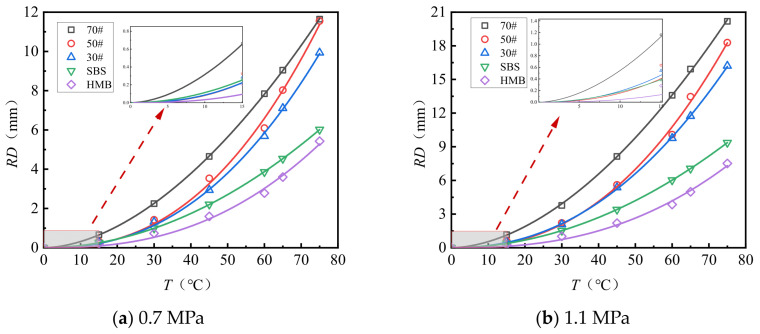
Temperature versus rutting deformation: curve fitting analysis (AC-16).

Figure 5 and Figure 6 illustrate the rutting deformation curves of AC-13 and AC-16 at various temperatures following 10,080 loading cycles applied at 0.7 MPa and 1.1 MPa. The observed deformation trends correspond closely to a power function. The experimental data were fitted and analyzed using Equation (2), yielding the fitting coefficients for temperature and rutting deformation, as presented in Table 9.

(2) $$RD=aP^{b}$$

where *RD* is the predicted rutting deformation, mm; *P* is the load magnitude, MPa; and *a* and *b* are the fitting coefficients.

As demonstrated in Figure 5 and Figure 6 and Table 9, the relationship between temperature and rutting deformation can be accurately modeled using a power function, with all fitting coefficients approaching 0.99. Under consistent gradation types and load magnitudes, this temperature–rutting relationship depends on the type of asphalt. The constant parameter “a” in the fitting equation shows an inverse correlation with the asphalt softening point, with values for 70# asphalt being 46 to 80 times greater than those for HMB asphalt. In contrast, the exponent “b” increases as the softening point rises, with HMB asphalt exhibiting values 1.4 to 1.5 times higher than those of 70# asphalt. Both parameters, “a” and “b”, significantly influence rutting behavior. At a test temperature of 90 °C under a load of 0.7 MPa, the predicted rut depth for 70# asphalt AC-13 is 29.2 mm, whereas HMB asphalt AC-13 demonstrates enhanced performance, with a predicted rut depth of only 16.3 mm, corresponding to 55.8% of the deformation observed in 70# asphalt.

### 5.3. An Investigation into the Impact of Load on the Rutting Depth of Asphalt Pavement Surface Layer Materials

**Figure 7:**
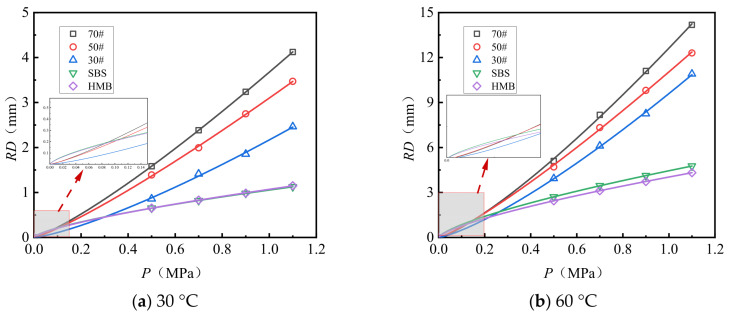
Load versus rut deformation fitting curve (AC-13).

**Figure 8:**
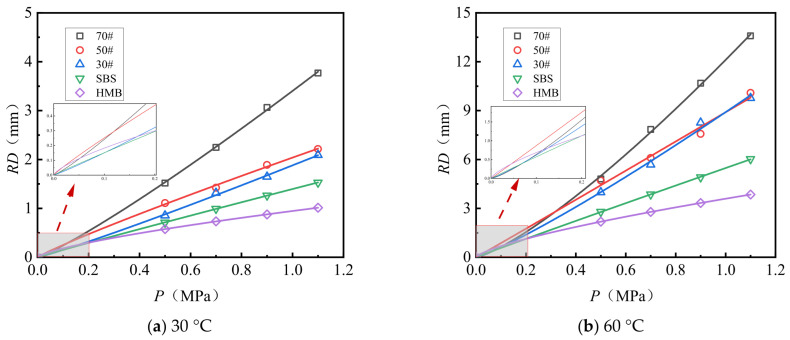
Load versus rut deformation fitting curve (AC-16).

Figure 7 and Figure 8 illustrate the rutting deformation curves of AC-13 and AC-16 following 10,080 load applications under varying load conditions. The observed deformation trends correspond closely to a power function. The experimental data were fitted and analyzed using Equation (2), yielding the fitting coefficients relating load to rutting deformation, which are presented in Table 10.

Analysis of Figure 7 and Figure 8 and Table 10 reveals the following observations: (1) The relationship between load and rut deformation can be accurately modeled using an exponential function, with fitting coefficients consistently approximating 0.97. (2) Under conditions of identical gradation type and test temperature, the load–rut deformation relationship is influenced by the asphalt type. Specifically, both the constant parameter (*a*) and the exponent (*b*) in the fitting function decrease as the asphalt’s softening point increases. Notably, compared to HMB asphalt, the constant a for 70# asphalt is approximately 3.1 to 3.6 times greater, while the exponent b is about 1.6 to 1.8 times higher. Assuming a test load of 1.5 MPa, representing an overload condition, the predicted rut depths for 70# asphalt AC-13 at test temperatures of 30 °C and 60 °C are 6.0 mm and 21.1 mm, respectively. In contrast, the predicted rut depths for HMB asphalt AC-13 under the same conditions are 1.4 mm and 5.4 mm, corresponding to only 23.3% and 25.6% of the rut depths observed for 70# asphalt.

### 5.4. An Investigation into the Impact of the Frequency of Load Applications on the Rutting Depth of Asphalt Pavement Surface Layer Materials

**Figure 9:**
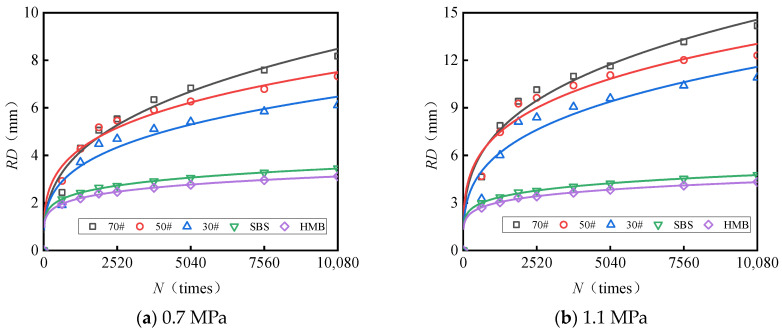
Fitting curve depicting the relationship between the number of loads and rut deformation (AC-13).

**Figure 10:**
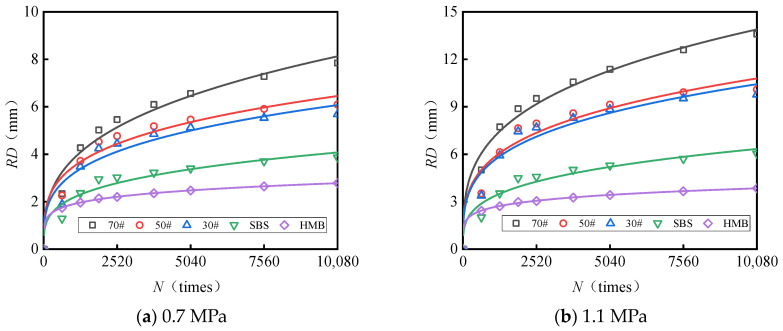
Fitting curve depicting the relationship between the number of loads and rut deformation (AC-16).

At a testing temperature of 60 °C, the correlation between the number of loading cycles and the rut depth for the AC-13 and AC-16 mixtures under varying load conditions (0.7 MPa and 1.1 MPa) is illustrated in Figure 9 and Figure 10. The observed trends in the experimental data exhibit a pattern consistent with a power function. The results were subsequently fitted and analyzed using Equation (2), yielding the fitting coefficients related to load and rut deformation, which are presented in Table 11.

Analysis of Figure 9 and Figure 10 and Table 11 reveals the following observations: (1) The relationship between the number of load cycles and rutting deformation can be accurately modeled using a power function, with correlation coefficients exceeding 0.86 in all cases. (2) When both the gradation type and load magnitude are held constant, the relationship between load repetitions and rut deformation is significantly affected by the asphalt type. Specifically, the fitting parameter a increases as the asphalt softening point rises; for HMB asphalt, the parameter a is 1.1 to 1.9 times greater than that of #70 asphalt. Conversely, the exponent b decreases with an increasing asphalt softening point; the exponent b for #70 asphalt is 1.8 to 2.1 times higher than that for HMB asphalt. Under the conditions of one million load applications, a test temperature of 60 °C, and a load of 0.7 MPa, the predicted rut depth for the AC-13 mixture containing #70 asphalt is 41 mm, whereas for AC-13 with HMB asphalt, the predicted rut depth is 7 mm, representing only 17% of the rut depth observed with #70 asphalt.

The observed power-law correlation between rut depth and variables such as temperature, load magnitude, and number of load repetitions can be mechanistically attributed to the progressive deterioration of the internal structure of the asphalt mixture. At the initial stages, characterized by a low number of load cycles, the rapid increase in rut depth predominantly results from densification (compaction) and minor rearrangements within the aggregate skeleton. As loading persists, the dominant deformation mechanism transitions to shear flow. Elevated temperatures, by inducing binder softening, as previously described, substantially diminish the shear strength of the asphalt mastic. Simultaneously, increased vertical loads generate higher shear stresses within the pavement layer. When the combined effect of reduced shear strength and elevated shear stress surpasses a critical threshold, the aggregate skeleton becomes destabilized, leading to particle displacement and a loss of interlocking. This synergistic interaction accelerates irreversible shear deformation, which corresponds to the stable rutting growth phase. The markedly lower rut depths observed in mixtures incorporating high-softening-point binders, such as SBS and HMB, under comparable conditions highlight their superior capacity to preserve mastic stiffness and aggregate interlock, thereby effectively resisting shear flow-induced deformation.

## 6. Temperature-Dependent Model for Rutting Depth of Asphalt Pavement Surface Layer Materials

Based on the analysis of the test results, it is evident that temperature, load, and the number of load applications are the primary factors affecting the rutting depth of the mixture, with their relationship to rutting depth following a power function. Consequently, the rutting prediction model for the mixture is proposed in Equation (3).

(3) $$RD=k_{0}P^{k_{p}}T^{k_{T}}N^{k_{N}}$$

where *RD* is the predicted rutting deformation, mm; *P* is the testing load magnitude, MPa; *T* is the testing temperature, °C; *N* is the loading number, times; and *k*_0_, *k_P_*, *k_T_*, and *k_N_* are regression coefficients.

Using Equation (3), the parameters of the rutting prediction model for the surface layer mixture were fitted, with the results shown in Table 12.

The data indicate a strong fit between the model and Equation (3), with correlation coefficients exceeding 0.95. The coefficient *k_P_*, representing the load index, varies among asphalt mixture types from 0.727 to 1.222; mixtures with higher asphalt softening points tend to have lower *k_P_* values. The temperature index *k_T_* ranges from 1.926 to 2.177, with mixtures containing asphalt of higher softening points exhibiting slightly higher *k_T_* values. The coefficient *k_N_*, related to the number of load applications, ranges from 0.133 to 0.295. Higher values of *k_P_*, *k_T_*, and *k_N_* indicate weaker resistance to rutting. The load variation ranges from approximately 0.7 to 1.5 MPa, the temperature variation spans roughly 15 to 80 °C, and the action variation range is nearly infinite. The *k_T_* value is slightly higher than that of asphalt with a high softening point (N), but the *k_P_* value is considerably lower than that of asphalt with a low softening point. During the initial loading phase, *k_P_* and *k_T_* have a greater impact on rutting depth. However, as the number of loads increases, the influence of *k_N_* gradually becomes more significant, indicating that the rutting depth of the HMB asphalt mixture is notably lower than that of the 70# asphalt mixture in the later stages of loading.

The parameters of the unified rutting model (*k_P_*, *k_T_*, *k_N_*) provide significant insights into the rutting resistance of asphalt mixtures from both rheological and mechanical perspectives. A reduction in the load index (*k_P_*) with an increasing asphalt softening point is attributable to the enhanced stiffness and elasticity of the binder. Mixtures containing stiffer mastics, such as those with SBS and HMB modifiers, demonstrate a more linear elastic response under load, resulting in deformation that is less sensitive to increasing stress levels and thus yielding *k_P_* values closer to unity. Conversely, softer base asphalts tend to exhibit nonlinear plastic flow and shear deformation, which accelerates rutting progression under load and corresponds to *k_P_* values exceeding one. The temperature index (*k_T_*), which is the largest among the indices, clearly indicates that temperature is the predominant factor influencing rutting behavior. The magnitude of *k_T_* reflects the temperature sensitivity of the binder’s viscosity, with a slight increase observed in modified asphalts likely due to the greater temperature dependence of their complex modulus within the high-temperature range tested. Lastly, the action index (*k_N_*), which decreases as binder performance improves, quantifies the mixture’s resistance to the accumulation of plastic deformation under repeated loading. The low *k_N_* values observed in modified asphalts align with their enhanced elastic recovery, which mitigates the net accumulation of permanent strain per load cycle. Collectively, these indices characterize the material’s performance, where reductions in *k_P_* and *k_N_* indicate a transition from viscous–plastic to more favorable elastic–solid behavior, a transition directly attributable to the high-softening-point and polymer-modified binders.

The advancement of predictive models for rutting in asphalt mixtures has progressed through a series of distinct methodological frameworks. Zhu et al. [29] enhanced indoor rutting prediction by utilizing parameters derived from the dynamic modulus, thereby establishing significant correlations between wheel tracking tests and full-scale accelerated testing. Wang et al. [30] applied grey relational analysis to systematically assess the influence of testing conditions—such as load, temperature, air void content, and moisture—on rut depth measurements obtained from the Asphalt Pavement Analyzer, leading to the development of a tailored prediction model specific to this device. Ziari et al. [31] focused on fundamental constitutive properties by formulating a flow number-based predictive model that mathematically links mix design parameters and gyratory compaction slope to dynamic creep test outcomes. Deng et al. [32] adopted two mechanistic–empirical models that were rigorously calibrated and validated through integrated laboratory testing under moving loads and finite element simulations. Complementing these methodologies, Ji et al. [33] advanced our theoretical understanding by introducing a second-order modified Burgers rheological model for rutting prediction.

Compared to existing methodologies, the present study offers distinct advantages through the development of a unified constitutive framework. While prior models have contributed valuable insights within their specific contexts, they frequently demonstrate limitations in concurrently addressing the interrelated effects of temperature, load magnitude, and loading cycles. The model proposed herein effectively integrates these three critical factors into a single constitutive formulation, thereby overcoming the fragmentation characteristic of earlier approaches. Moreover, the incorporation of the Boltzmann function facilitates a more physically meaningful representation of the temperature-dependent behavior of dynamic stability, surpassing the descriptive capacity of traditional empirical fittings. Furthermore, although the model parameters have well-defined physical interpretations, their estimation relies on standardized laboratory tests, which may impose certain constraints on their applicability in engineering applications [34]. This integrated methodology not only improves predictive accuracy but also affords a clearer mechanistic understanding of rutting phenomena while preserving practical applicability through parameters that can be obtained via standard laboratory testing. Consequently, the framework constitutes a significant advancement in reconciling theoretical rigor with engineering practicality in the evaluation of asphalt mixture performance.

The practical implementation of the integrated rutting prediction model requires further enhancement. While the proposed framework demonstrates validity within controlled laboratory settings, its verification across a wider spectrum of field conditions remains insufficient, particularly concerning real-world factors such as environmental aging and moisture influences. Moreover, although the model parameters are founded on well-defined physical principles, their calibration predominantly depends on standardized laboratory testing procedures, which may limit their applicability in field scenarios. Notably, the exclusive reliance on the wheel tracking test—despite its standardization and direct relevance to rutting performance—restricts a comprehensive evaluation of the material’s full viscoelastic properties. Furthermore, the maximum experimental temperature of 75 °C does not encompass higher temperature extremes that may occur in severe climatic conditions, thereby limiting the model’s practical relevance.

## 7. Conclusions

This research, grounded in controlled indoor rutting experiments, examined the dynamic stability and key influencing factors of asphalt pavement surface layer materials. Furthermore, it proposed a temperature-dependent model to describe the high-temperature performance of these materials, alongside a predictive model for rutting behavior. The principal findings of this study are summarized as follows:

(1) The investigation into the impact of temperature on the dynamic stability of asphalt pavement surface layer materials revealed that dynamic stability diminishes with increasing temperature, following an S-shaped curve. Notably, the dynamic stability at 15 °C exceeds that at 75 °C by more than an order of magnitude. At lower temperatures, dynamic stability is more sensitive to variables such as applied load and nominal maximum particle size. Specifically, at 15 °C, an increase in load results in a significant decline in dynamic stability, whereas at 75 °C, mixtures with identical asphalt types and mineral aggregate gradations exhibit dynamic stability values that tend to plateau.

(2) A temperature-dependent dynamic stability model was developed utilizing the Boltzmann function, effectively capturing the relationship between dynamic stability and temperature. The observed decrease in dynamic stability with rising temperature follows a three-phase pattern characterized as “slow–fast–slow.” The transition temperatures identified for the dynamic stability models of 70#, 50#, 30#, SBS, and HMB asphalt mixtures are 39 °C, 44 °C, 46 °C, 56 °C, and 56 °C, respectively.

(3) This study further explored the determinants of rut depth in asphalt pavement surface layer materials and established a predictive model for rutting. The analysis demonstrated that rut deformation increases with temperature (*T*), applied load (*P*), and the number of load repetitions (*N*). By introducing indices for load (*k_P_*), temperature (*k_T_*), and load frequency (*k_N_*), rut prediction models were developed for asphalt surface layer mixtures under diverse operational conditions. These indices range from 0.727 to 1.222 for *k_P_*, 1.926 to 2.177 for *k_T_*, and 0.133 to 0.295 for *k_N_*. The models exhibit correlation coefficients exceeding 0.95, indicating a high level of accuracy and robustness in predicting rut depth in asphalt mixtures.

(4) The transition temperatures (e.g., 56 °C for SBS/HMB versus 39 °C for 70#) offer a clear, quantitative basis for selecting asphalt binders tailored to specific regional climate conditions. The rutting prediction model and its indices (*k_P_*, *k_T_*, *k_N_*) can be integrated into mechanistic–empirical pavement design methods to forecast rutting depth under various combinations of traffic load and temperature. This integration aids in optimizing pavement layer thickness and planning long-term maintenance schedules.

Future work will systematically address the current limitations through three primary avenues. Firstly, dynamic modulus testing and repeated creep testing will be utilized to thoroughly characterize the full viscoelastic properties of asphalt mixtures, thereby providing a more comprehensive understanding beyond the findings obtained from the existing wheel tracking tests. Secondly, extended monitoring of pavement performance will provide a dependable field validation dataset to support subsequent model verification. Thirdly, temperature-controlled testing protocols will be extended to 80 °C and higher in order to directly assess the performance of mixtures under extreme thermal conditions.

## Figures and Tables

**Table 1:** Technical properties of base asphalt.

Test Item		70#		50#		30#	
		Test Value	Required Value	Test Value	Required Value	Test Value	Required Value
Penetration at 25 °C (0.1 mm)		69	60–80	52	40–60	35	20–40
Penetration index, PI		−0.654	−1.5–1.0	−0.385	−1.5–1.0	−0.083	−1.5–1.0
10 °C ductility (cm)		38	≥20	21	≥15	12	≥10
Softening point (°C)		48	≥46	53	≥49	59	≥55
15 °C relative density		1.018	/	1.009	/	1.021	/
Rotating film aging test	Percentage change in quality (%)	−0.215	≤±0.8	−0.337	≤±0.8	−0.392	≤±0.8
	Penetration ratio at 25 °C (%)	69	≥61	65	≥63	68	≥65
	10 °C ductility (cm)	8	≥6	6	≥4	/	/

**Table 2:** Properties of SBS-modified asphalt and HMB high-modulus asphalt technology.

Test Item		SBS		HMB	
		Test Value	Required Value	Test Value	Required Value
Penetration at 25 °C (0.1 mm)		47	30–60	29	20–35
Penetration index (PI)		0.239	≥0	/	/
5 °C ductility (cm)		38	≥20	/	/
25 °C ductility (cm)		/	/	46	≥25
Softening point (°C)		75	≥60	83	≥60
Elastic recovery at 25 °C (%)		96	≥75	63	≥55
15 °C relative density		1.025	/	1.031	/
Rotating film aging test	Percentage change in quality (%)	−0.173	≤±1.0	−0.086	≤±0.5
	Penetration ratio at 25 °C (%)	72	≥65	76	≥65

**Table 3:** Technical properties of coarse aggregates.

Test Item	The Following Are the Test Values of Technical Properties of Coarse Aggregates (mm)				Required Value
	16–19	13.2–16	9.5–13.2	4.75–9.5	
Apparent relative density	2.837	2.825	2.820	2.818	≥2.6
Absorption rate (%)	0.34	0.40	0.47	0.68	≤2
Content of needle-like and flake-like particles (%)	7.5	6.2	5.1	3.2	≤15
Crushing value (%)	16.7				≤26
Los Angeles abrasion loss (%)	18.1				≤28
Adhesion to asphalt	5				≥4

**Table 4:** Fine aggregate technical properties.

Test Item	Test Value	Required Value
Apparent relative density	2.742	≥2.5
Sturdiness (%)	8.4	≤12
Methylene blue value (g/kg)	3.3	≤25
Angularity (s)	43.8	≥30

**Table 5:** Technical properties of the mineral powder.

Test Item		Test Value	Required Value
Apparent relative density		2.738	≥2.50
Granularity range (%)	<0.6 mm	100	100
	<0.15 mm	99.6	90–100
	<0.075 mm	97.2	75–100
Hydrophilic coefficient		0.6	<1
Plasticity index (%)		3.0	<4

**Table 6:** Relative values of the maximum dynamic stability *DS_max_* of surface layer materials.

Type of Mixture	Asphalt Type	The Relative Values of *DS_max_* of Surface Layer Materials Under the Following Loads (MPa)			
		0.5	0.7	0.9	1.1
AC-13	70#	1.61	1.49	1.24	1.00
	50#	1.39	1.29	1.19	1.00
	30#	1.34	1.25	1.17	1.00
	SBS	1.23	1.12	1.06	1.00
	HMB	1.19	1.09	1.05	1.00
AC-16	70#	1.54	1.43	1.22	1.00
	50#	1.35	1.26	1.18	1.00
	30#	1.32	1.24	1.16	1.00
	SBS	1.22	1.11	1.06	1.00
	HMB	1.18	1.09	1.05	1.00

**Table 7:** Summary of the coefficient of variation for dynamic stability.

Type of Mixture	Asphalt Type	Temperature (°C)	The Relative Values of *DS_max_* Under the Following Loads (MPa)			
			0.5	0.7	0.9	1.1
AC-13	70#	0	2.6%	1.6%	4.4%	3.0%
		30	7.7%	3.9%	5.7%	4.7%
		45	1.3%	3.1%	0.5%	4.6%
		60	5.5%	3.6%	6.1%	1.0%
		65	0.8%	4.4%	6.6%	3.5%
		75	1.5%	0.6%	5.5%	5.3%
	50#	0	1.2%	2.3%	4.2%	1.3%
		30	0.5%	3.7%	7.5%	7.9%
		45	5.6%	7.3%	1.8%	7.3%
		60	7.4%	0.5%	0.2%	7.7%
		65	4.3%	0.8%	4.0%	7.2%
		75	4.8%	0.7%	2.8%	1.1%
	30#	0	1.3%	7.4%	1.4%	3.8%
		30	1.0%	6.9%	3.0%	7.4%
		45	7.3%	1.9%	6.2%	0.6%
		60	2.1%	4.7%	2.5%	6.4%
		65	6.3%	5.4%	1.8%	5.2%
		75	7.9%	6.9%	0.2%	7.9%
	SBS	0	6.6%	3.8%	3.0%	6.3%
		30	2.8%	3.3%	5.4%	7.1%
		45	1.6%	2.8%	5.2%	1.8%
		60	3.6%	6.3%	5.0%	5.0%
		65	5.5%	3.0%	0.5%	1.6%
		75	2.7%	5.4%	1.3%	3.9%
	HMB	0	4.2%	3.6%	5.6%	4.7%
		30	4.4%	2.9%	7.0%	0.4%
		45	6.5%	4.7%	0.3%	0.4%
		60	5.5%	1.3%	0.6%	4.4%
		65	0.7%	5.6%	3.8%	3.2%
		75	6.3%	4.2%	5.5%	3.8%
AC-16	70#	0	7.2%	2.9%	7.7%	6.8%
		30	5.8%	2.6%	1.7%	1.9%
		45	5.1%	0.3%	4.6%	7.1%
		60	0.1%	7.5%	3.4%	6.4%
		65	3.9%	1.2%	4.4%	2.3%
		75	1.1%	0.7%	6.2%	7.8%
	50#	0	7.3%	0.7%	0.9%	7.9%
		30	7.5%	7.8%	4.4%	6.9%
		45	6.5%	6.5%	1.6%	1.4%
		60	5.6%	8.0%	3.7%	6.9%
		65	4.9%	4.2%	1.1%	4.3%
		75	6.2%	0.8%	1.3%	6.3%
	30#	0	7.0%	6.8%	1.2%	2.6%
		30	4.6%	4.7%	5.7%	6.0%
		45	5.2%	2.3%	7.3%	3.2%
		60	1.8%	7.9%	7%	6.8%
		65	4.9%	4.5%	5.4%	4.3%
		75	6.5%	3.6%	7.5%	0.1%
	SBS	0	3.2%	1.0%	7.4%	7.3%
		30	7.6%	7.3%	7.9%	0.5%
		45	5.3%	3.7%	3.7%	1.3%
		60	5.6%	2.7%	0.1%	0.4%
		65	5.6%	2.2%	6.9%	6.0%
		75	6.7%	6.4%	6.3%	6.7%
	HMB	0	4.1%	3.7%	8%	1.7%
		30	0.6%	1.8%	6.3%	1.4%
		45	6.1%	5.8%	6.5%	8.0%
		60	3.8%	7.7%	4.1%	7.3%
		65	5.5%	6.9%	3.8%	2.8%
		75	7.6%	3.8%	5.8%	0.4%

**Table 8:** Fitting coefficients of the temperature-dependent model for dynamic stability.

Type of Mixture	Type of Asphalt	The Following Loads (MPa) the Lower Surface Layer Material *DS_max_* (times/mm) *T*_0_ (°C)							
		*DS_max_*				*T*_0_			
		0.5	0.7	0.9	1.1	0.5	0.7	0.9	1.1
AC-135	70#	12,598	11,649	9750	7854	39.3	39.3	39.1	38.9
	50#	13,211	12,285	11,361	9512	44.7	44.7	44.6	44.5
	30#	15,261	14,305	13,349	11,437	46.8	46.7	46.7	46.6
	SBS	21,168	19,168	18,169	17,169	56.4	56.4	56.4	56.4
	HMB	25,532	23,502	22,487	21,472	55.9	55.9	55.9	55.9
AC-16	70#	13,658	12,705	10,800	8896	39.5	39.4	39.3	39.1
	50#	14,145	13,223	12,300	10,456	45.8	45.8	45.7	45.7
	30#	15,935	14,971	14,009	12,088	47.0	47.0	47.0	46.9
	SBS	22,091	20,089	19,089	18,088	56.5	56.5	56.5	56.5
	HMB	26,592	24,554	23,534	22,526	56.2	56.2	56.2	56.2

**Table 9:** Regression coefficients for the relationship between temperature and rut deformation.

Type of Mixture	Load (MPa)	Type of Asphalt	*a*	*b*	*R* ^2^
AC-13	0.7	70#	0.00958	1.783	0.99
		50#	0.00633	1.849	0.99
		30#	0.00242	2.048	0.99
		SBS	0.00005	2.821	0.99
		HMB	0.00012	2.588	0.99
	1.1	70#	0.00605	1.760	0.99
		50#	0.00321	1.886	0.99
		30#	0.00054	2.278	0.99
		SBS	0.00004	2.816	0.99
		HMB	0.00009	2.590	0.99
AC-16	0.7	70#	0.00511	1.791	0.99
		50#	0.00030	2.442	0.99
		30#	0.00038	2.355	0.99
		SBS	0.00122	1.969	0.99
		HMB	0.00011	2.504	0.99
	1.1	70#	0.00873	1.795	0.99
		50#	0.00067	2.365	0.99
		30#	0.00119	2.203	0.99
		SBS	0.00183	1.979	0.99
		HMB	0.00014	2.509	0.99

**Table 10:** Coefficients of the regression model relating load to rut deformation.

Type of Mixture	Temperature (°C)	Type of Asphalt	*a*	*b*	*R* ^2^
AC-13	30	70#	3.674	1.214	0.99
		50#	3.099	1.183	0.99
		30#	2.169	1.298	0.99
		SBS	1.054	0.696	0.99
		HMB	1.070	0.716	0.99
	60	70#	12.625	1.266	0.99
		50#	11.041	1.192	0.99
		30#	9.595	1.292	0.99
		SBS	4.449	0.716	0.99
		HMB	4.030	0.721	0.99
AC-16	30	70#	3.402	1.148	0.99
		50#	2.039	0.909	0.99
		30#	1.880	1.097	0.99
		SBS	1.396	0.966	0.99
		HMB	0.947	0.715	0.99
	60	70#	12.112	1.278	0.99
		50#	8.899	1.009	0.97
		30#	8.905	1.156	0.98
		SBS	5.476	0.979	0.99
		HMB	3.594	0.718	0.99

**Table 11:** Correlation coefficient between the number of actions and rutting deformation.

Type of Mixture	Load (MPa)	Type of Asphalt	*a*	*b*	*R* ^2^
AC-13	0.7	70#	0.359	0.343	0.95
		50#	0.621	0.270	0.94
		30#	0.451	0.289	0.86
		SBS	0.734	0.168	0.99
		HMB	0.669	0.167	0.99
	1.1	70#	0.825	0.311	0.93
		50#	1.104	0.268	0.89
		30#	0.708	0.303	0.86
		SBS	1.030	0.166	0.99
		HMB	0.934	0.166	0.99
AC-16	0.7	70#	0.384	0.331	0.93
		50#	0.533	0.270	0.88
		30#	0.453	0.281	0.86
		SBS	0.323	0.275	0.86
		HMB	0.597	0.167	0.99
	1.1	70#	0.878	0.299	0.96
		50#	0.842	0.277	0.86
		30#	0.817	0.276	0.86
		SBS	0.458	0.285	0.87
		HMB	0.842	0.165	0.99

**Table 12:** Fitting parameters of the rutting prediction model for surface layer mixtures.

Mixture Type	Asphalt Type	*k*_0_	*k_P_*	*k_T_*	*k_N_*	*R* ^2^
AC-13	HMB	0.0002	0.733	2.133	0.142	0.981
	SBS	0.0002	0.728	2.177	0.133	0.974
	30#	0.0002	1.136	2.166	0.203	0.975
	50#	0.0002	1.128	2.066	0.268	0.983
	70#	0.0002	1.206	2.068	0.287	0.961
AC-16	HMB	0.0003	0.733	2.011	0.137	0.979
	SBS	0.0003	0.727	2.044	0.144	0.951
	30#	0.0003	1.121	2.068	0.201	0.975
	50#	0.0003	1.095	2.022	0.235	0.971
	70#	0.0003	1.222	1.926	0.295	0.992

## Data Availability

The original contributions presented in this study are included in this article. Further inquiries can be directed to the corresponding author.
